# Deep learning models for hepatitis E incidence prediction leveraging meteorological factors

**DOI:** 10.1371/journal.pone.0282928

**Published:** 2023-03-13

**Authors:** Yi Feng, Xiya Cui, Jingjing Lv, Bingyu Yan, Xin Meng, Li Zhang, Yanhui Guo

**Affiliations:** 1 Shandong Provincial Key Laboratory of Infectious Disease Control and Prevention, Shandong Center for Disease Control and Prevention, Jinan, Shandong, China; 2 School of Data and Computer Science, Shandong Women’s Unversity, Jinan, Shandong, China; 3 School of Public Health, Shandong University, Jinan, Shandong, China; Wuhan University of Technology, CHINA

## Abstract

**Background:**

Infectious diseases are a major threat to public health, causing serious medical consumption and casualties. Accurate prediction of infectious diseases incidence is of great significance for public health organizations to prevent the spread of diseases. However, only using historical incidence data for prediction can not get good results. This study analyzes the influence of meteorological factors on the incidence of hepatitis E, which are used to improve the accuracy of incidence prediction.

**Methods:**

We extracted the monthly meteorological data, incidence and cases number of hepatitis E from January 2005 to December 2017 in Shandong province, China. We employ GRA method to analyze the correlation between the incidence and meteorological factors. With these meteorological factors, we achieve a variety of methods for incidence of hepatitis E by LSTM and attention-based LSTM. We selected data from July 2015 to December 2017 to validate the models, and the rest was taken as training set. Three metrics were applied to compare the performance of models, including root mean square error(RMSE), mean absolute percentage error(MAPE) and mean absolute error(MAE).

**Results:**

Duration of sunshine and rainfall-related factors(total rainfall, maximum daily rainfall) are more relevant to the incidence of hepatitis E than other factors. Without meteorological factors, we obtained 20.74%, 19.50% for incidence in term of MAPE, by LSTM and A-LSTM, respectively. With meteorological factors, we obtained 14.74%, 12.91%, 13.21%, 16.83% for incidence, in term of MAPE, by LSTM-All, MA-LSTM-All, TA-LSTM-All, BiA-LSTM-All, respectively. The prediction accuracy increased by 7.83%. Without meteorological factors, we achieved 20.41%, 19.39% for cases in term of MAPE, by LSTM and A-LSTM, respectively. With meteorological factors, we achieved 14.20%, 12.49%, 12.72%, 15.73% for cases, in term of MAPE, by LSTM-All, MA-LSTM-All, TA-LSTM-All, BiA-LSTM-All, respectively. The prediction accuracy increased by 7.92%. More detailed results are shown in results section of this paper.

**Conclusions:**

The experiments show that attention-based LSTM is superior to other comparative models. Multivariate attention and temporal attention can greatly improve the prediction performance of the models. Among them, when all meteorological factors are used, multivariate attention performance is better. This study can provide reference for the prediction of other infectious diseases.

## Introduction

Viral hepatitis is recognized as a major public health problem and is now considered to be comparable with the three major infectious diseases(AIDS, malaria, and tuberculosis) [1]. Hepatitis E virus (HEV) is the most common cause of acute viral hepatitis worldwide, leading to a major public health problem [2]. HEV infection mainly breaks out in developing and industrialized countries, especially in Asia, Africa and Central America [3]. There are around 20 million cases worldwide, with approximately 3.3 million symptomatic cases, leading to 55,000 HEV-related deaths annually. The fatality rate in young adults reached 0.5–3%.HEV infection has a poor prognosis among pregnant woman, especially in the third trimester, where the fatality rate can reach up to 30% [4–6]. Sporadic hepatitis E has caused over 50% of acute viral hepatitis cases in recent years [7], which caused the huge social, economic, and health burden. To better mitigate future outbreaks, a method is needed to accurately predict the incidence of hepatitis E. US Centers for Disease Control and Prevention have openly endorsed adopting models to inform decision making [8].

It is relatively well accepted that climate change can affect human pathogenic diseases. Camilo Mora indicated that over half of known human pathogenic diseases can be aggravated by climate change [9]. Li et al. [10] studied the relationship between the epidemic of SARS, dengue, influenza, respiratory syncytial virus and meteorological factors, and believed that specific meteorological factors were the driving force of the virus epidemic. A large number of literature show that meteorological factors are related to hand foot mouth disease [11, 12], COVID-19 [13, 14] and other diseases. Some researchers suggested that rainfall has a certain impact on the spread of hepatitis A virus [15]. Kiook Baek [16] analyzed the association between temperature and precipitation and the incidence of hepatitis A in Seoul, which proved that meteorological factors have an impact on hepatitis A and are helpful to predict the incidence of hepatitis A. For hepatitis E, Anna investigated that population density and water balance influence the global occurrence of hepatitis E epidemics. Vianney [17] showed that hepatitis E outbreak associated with rainfall in the Central African Republic. Understanding which meteorological factors have a greater impact on the incidence of hepatitis E is conducive to effective prevention and treatment of hepatitis E. In this paper, we analyzed the incidence data and meteorological data of hepatitis E in the past 13 years, and reached an effective conclusion.

Besides, exploring the rules of historical data and predicting the future incidence of hepatitis E can achieve the purpose of accurate prevention and control. Time series methods are commonly used to solve the above problems. The traditional prediction models are the Autoregressive Integrated Moving Average (ARIMA) and its variants, including Seasonal ARIMA (SARIMA), ARIMAX. The ARIMA model, as a statistical regression model, is widely used to predict the incidence of various diseases, such as influenza [18], AIDS [19], COVID-19 [20]. However, the result might be unsatisfactory due to linear assumption requirements. Another mainstream to analyze time series is utilized by artificial intelligence methods, such as Markov model [21], artificial neural network [22], support vector machine(SVM) [23], etc. Among them, SVM model has been successfully used in many fields of time series prediction, including financial prediction [24] and disease prediction [25, 26], due to the generalization ability and nonlinear regression estimation. At present, benefiting from the powerful feature representation capabilities of deep learning, Recurrent Neural Network(RNN) [27] is an effective approach to analyze temporal data. Subsequently, LSTM, overcoming the lack of vanishing gradients in the RNN, was widely used in various fields, including disease prediction [28], energy consumption forecasting [29], solar power forecasting [30], oil markets prediction [31] and so on. Recently, some researchers have applied transformer to predict wind speed [32]. For hepatitis E incidence prediction, our previous work [25] adopted ARIMA, SVM, LSTM methods to predict hepatitis E incidence, obtaining the state of the art result by LSTM. Later, Xiaoqing Cheng et al. [33] utilized a variant of LSTM(Bi-LSTM) to predict hepatitis E incidence in Jiangsu, China.

In order to further improve the prediction accuracy, researchers used multiple factors to predict disease incidence. Some researchers leverage Google search index to improve the accuracy of disease prediction, such as influenza [34, 35], COVID-19 [36, 37] etc. Meteorological factors, as another important aspect to improve the performance of disease prediction, have been widely studied. A large number of literature have proved that the combination of meteorological factors and machine learning algorithms is conducive to improving the accuracy of disease incidence prediction, including dengue fever [38, 39], hand-foot-mouth disease [40], mumps [41] etc. For hepatitis E incidence prediction, Tu Peng et al. [42] demonstrated that meteorological factors (radiation, air pressure, precipitation) can contribute to the prediction effectiveness. Xiaoqing Cheng et al. [33] adopted Bi-LSTM model with the meteorological factors of temperature, rainfall to improve the prediction accuracy of hepatitis E incidence. However, Bi-LSTM model treats all factors equally, which will cause data with weak correlation to interfere with the model. In order to make full use of meteorological factors, we explore attention-based LSTM models with multi-factors, which can make different factors have different contributions to the models.

In this study, we adopt grey correlation model to analyze the importance of multiple meteorological factors with hepatitis E incidence. Then, LSTM and attention-based LSTMs are used to predict hepatitis E monthly incidence with and without meteorological factors, respectively. Experiments show that our proposed method obtains state-of-the-art performance. The construction and conclusions of models provide some references for the prevention and control of hepatitis E. Meanwhile, these methods are general and could also be suitable for predicting other diseases. The main contributions are listed as follows.

1. Grey correlation model is employed to analyze the correlation between hepatitis E incidence and meteorological factors. The conclusion is beneficial to the prevention and control of hepatitis E.
2. We propose several methods for predicting the incidence of hepatitis E, including MA-LSTM, TA-LSTM, BiA-LSTM. The performance of these methods are compared with other methods, significantly improving the prediction accuracy of hepatitis E.
3. The conclusion that attention mechanism and multiple factors can improve prediction accuracy of hepatitis E, provides a reference for other prediction problems.

## Materials and methods

### Materials source

This study collected information regarding hepatitis E from January 2005 to December 2017 in Shandong Province, China. Data were provided by the Shandong Center for Disease Control and Prevention, mainly including monthly incidence and monthly cases number of hepatitis E in Shandong. The meteorological data is abstracted from the China meteorological data sharing service system, which contains the statistical data of many meteorological stations. We take the average value of the meteorological data observed by each station as the provincial value of monthly meteorological data.

### Grey relational analysis

Grey relational analysis(GRA) is a multi-factor statistical analysis method to explore the similarity and dissimilarity among factors. GRA judges the relationship between different sequences by computing the similarity of the geometric shape of the sequence curve. It uses the grey relational grade to measure the relational degree of factors. First, we select the reference series and alternative series in all series. The reference series can express the behavior characteristics of the system, similar to the dependent variable. Alternative series is the factor that affects the reference series, similar to independent variable. In this study, monthly incidence of hepatitis E is regarded as reference series, and meteorological factors are taken as alternative series. Then, the series needs to be normalized to make the values free of unit. This process is called grey relational generating. We normalize all the series to [0, 1] by min-max normalization.

The next step is to calculate the grey relational coefficient(GRC), which is an indicator of the similarity between the reference series and alternatives series. The principle is shown in the formula (1)–(3). Where, y denotes the grey relational coefficient. a,b denote the extremum of the matrix. *x*_0_ denotes the reference series. *ρ* denotes the discrimination coefficient, set to 0.5.

$$\begin{array}{c} a=\underset{i}{\text{min}}\underset{k}{\text{min}}\left|x_{0k}-x_{ik}\right|,i=1,\cdots ,12;k=1,\cdots ,156 \end{array}$$ (1)

$$\begin{array}{c} b=\underset{i}{\text{max}}\underset{k}{\text{max}}\left|x_{0k}-x_{ik}\right|,i=1,\cdots ,12;k=1,\cdots ,156 \end{array}$$ (2)

$$\begin{array}{c} y\left(x_{0k},x_{ik}\right)=\frac{a+\rho b}{|x_{0k}-x_{ik}|+\rho b} \end{array}$$ (3)

Fially, the grey relational grade(GRG) is calculated by GRC for evaluating all the alternatives, as shown in Eq (4). A large GRG indicates a higher correlation with the reference series.

$$\begin{array}{c} y\left(x_{0},x_{i}\right)=\frac{1}{N}\sum_{k=1}^{N}y\left(x_{0k},x_{ik}\right),N=12 \end{array}$$ (4)

### LSTM model

**Fig 1:**
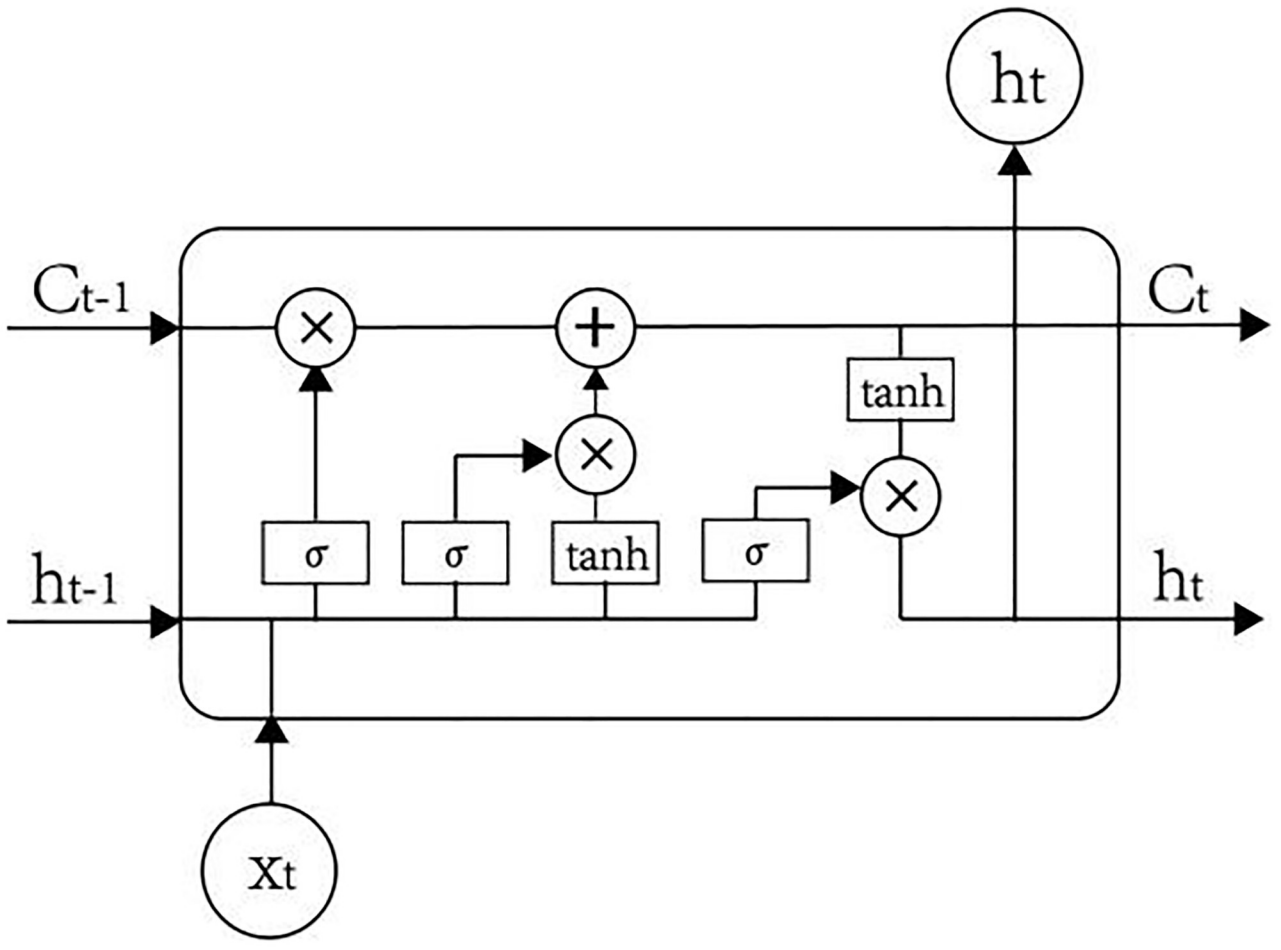
Structrue of LSTM cell.

LSTM is the most popular variant of RNN, which can solve the problem of long-term dependency and is suitable for processing and predicting time series. Different from RNN, LSTM consists of the memory cells to replace the hidden layer neurons. The state of memory cells are controlled by gate structure, including input gate, forget gate and output gate. These gates, as information filters, determine which information needs to be retained or ignored. Fig 1 shows the structure of LSTM memory cells. Assuming that the cell is at a state of t, the calculation is as follows.

$$\begin{array}{c} f_{\left(t\right)}=\sigma \left(W_{f}x_{\left(t\right)}+U_{f}h_{\left(t-1\right)}+b_{f}\right) \end{array}$$ (5)

$$\begin{array}{c} i_{\left(t\right)}=\sigma \left(W_{i}x_{\left(t\right)}+U_{i}h_{\left(t-1\right)}+b_{i}\right) \end{array}$$ (6)

$$\begin{array}{c} o_{\left(t\right)}=\sigma \left(W_{o}x_{\left(t\right)}+U_{o}h_{\left(t-1\right)}+b_{o}\right) \end{array}$$ (7)

$$\begin{array}{c} c_{\left(t\right)}=f_{\left(t\right)}⊗c_{\left(t-1\right)}+i_{\left(t\right)}⊗tanh\left(W_{c}x_{\left(t\right)}+U_{c}h_{\left(t-1\right)}+b_{c}\right) \end{array}$$ (8)

$$\begin{array}{c} h_{\left(t\right)}=o_{\left(t\right)}⊗tanh\left(c_{\left(t\right)}\right) \end{array}$$ (9)

The forget gate *f*_(*t*)_ controls how much information of the last cells state *c*_(*t*−1)_ is ignored. The input gate *i*_(*t*)_ indicates how much the current input information is retained to the state of *c*_(*t*)_. The output gate *o*_(*t*)_ controls to generates output result *h*_(*t*)_ according to the current state, which should be transferred to the next memory cell. Among the formula (5)–(9), *W*, *U* denote the weight matrix to be optimized, *b* represents the bias term. The symbol *σ* denotes the sigmoid activation function, ⊗ is the dot product operation.

#### Model for hepatitis E prediction

**Fig 2:**
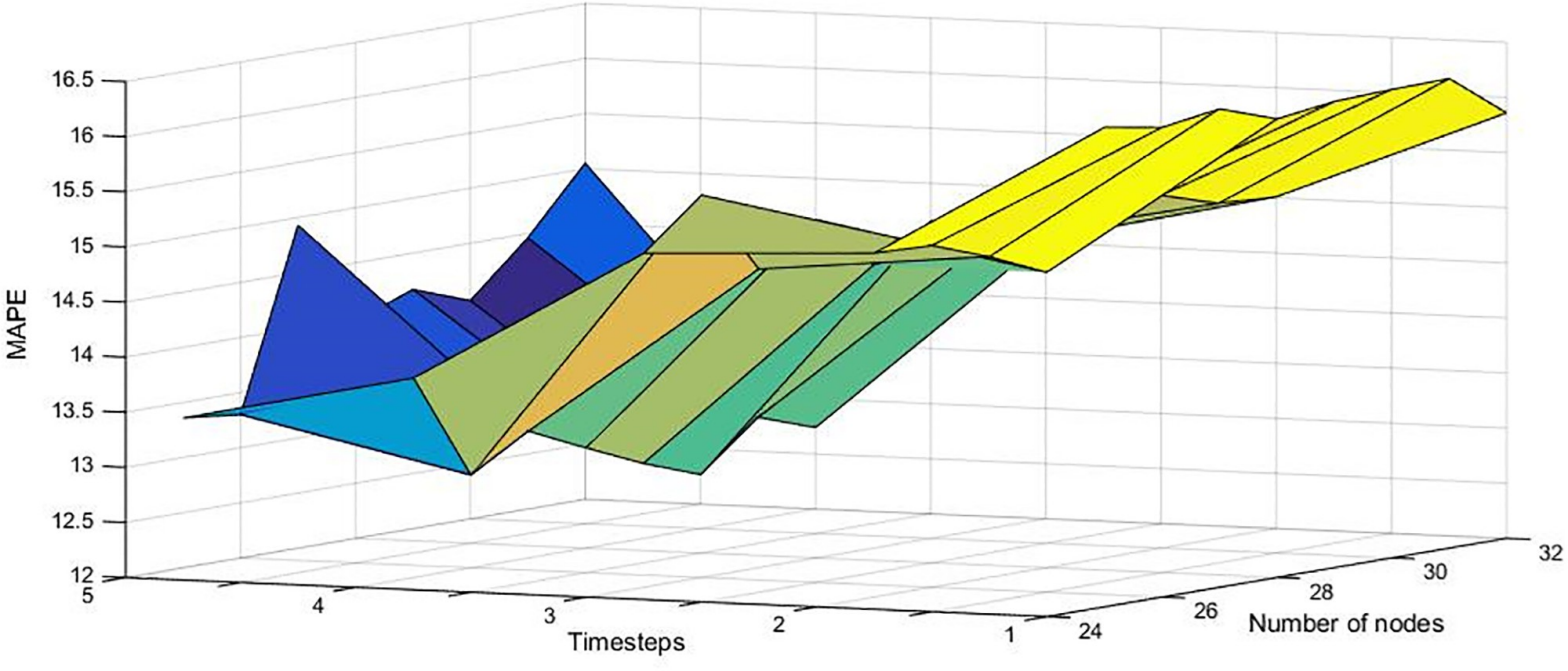
Results of grid search for LSTM.

In this study, we adopt LSTM model to implement incidence prediction of hepatitis E, by univariate and multivariate, respectively. Univariate method only employs the previous four monthly data(monthly incidence, monthly cases) of hepatitis E to predict the next monthly data. Multivariate method employs the previous four monthly data and multiple meteorological factors(temperature data, rainfall data, photoperiod data, etc). The form of input and output is shown as formula (10), according time step. ${\bar{x}}_{\left(t\right)}$, is the result that the model want to obtain. *x*_(*i*)_ is a number of monthly data or a vector of multi-factor data, for two methods, respectively. In order to design the network structure of LSTM, we employ the grid search method to carry out our experiments. The time step ranges from 1 to 5, and the number of nodes changes from 25 to 32. The experimental results show that the performance is the best when time step is set to 4 and the number of nodes is set to 30, as shown in Fig 2. The number of nodes in hidden layer of LSTM is 30. And the number of input layer nodes is 1 or 11 for univariate and multivariate methods. The output layer has 1 nodes for two methods.

$$\begin{array}{c} f_{X}\left(x_{\left(t-4\right)}\to x_{\left(t-3\right)}\to x_{\left(t-2\right)}\to x_{\left(t-1\right)}\right)\Rightarrow \left({\bar{x}}_{\left(t-3\right)}\to {\bar{x}}_{\left(t-2\right)}\to {\bar{x}}_{\left(t-1\right)}\to {\bar{x}}_{\left(t\right)}\right) \end{array}$$ (10)

#### Parameters setting of LSTM

To overcome the over-fitting problem of small samples, we employ dropout and regularization strategies. Dropout rate is set to 0.15, and regularization parameter is set to 0.001 in our models. The epochs for training is set to 220. Besides, we adopt Adam as the optimizer which is faster and better than stochastic gradient descent (SGD) method. Learn rate is set to 0.001.

### Attention-based LSTM

Attention mechanism, derived from the research of human vision, is to improve the attention to the key local features and ignore the useless information. By introducing the attention mechanism, deep learning models can solve the problem of information overload and improve the efficiency and performance. Firstly, the model needs to calculate the attention distribution by softmax function. Then, the input information is weighted according to the attention. For LSTM model, attention mechanism can be added to the time step to make the model focus on the time context. Attention mechanism can also be added to multivariables, so that the model can distinguish the importance of input information.

#### Temporal attention

**Fig 3:**
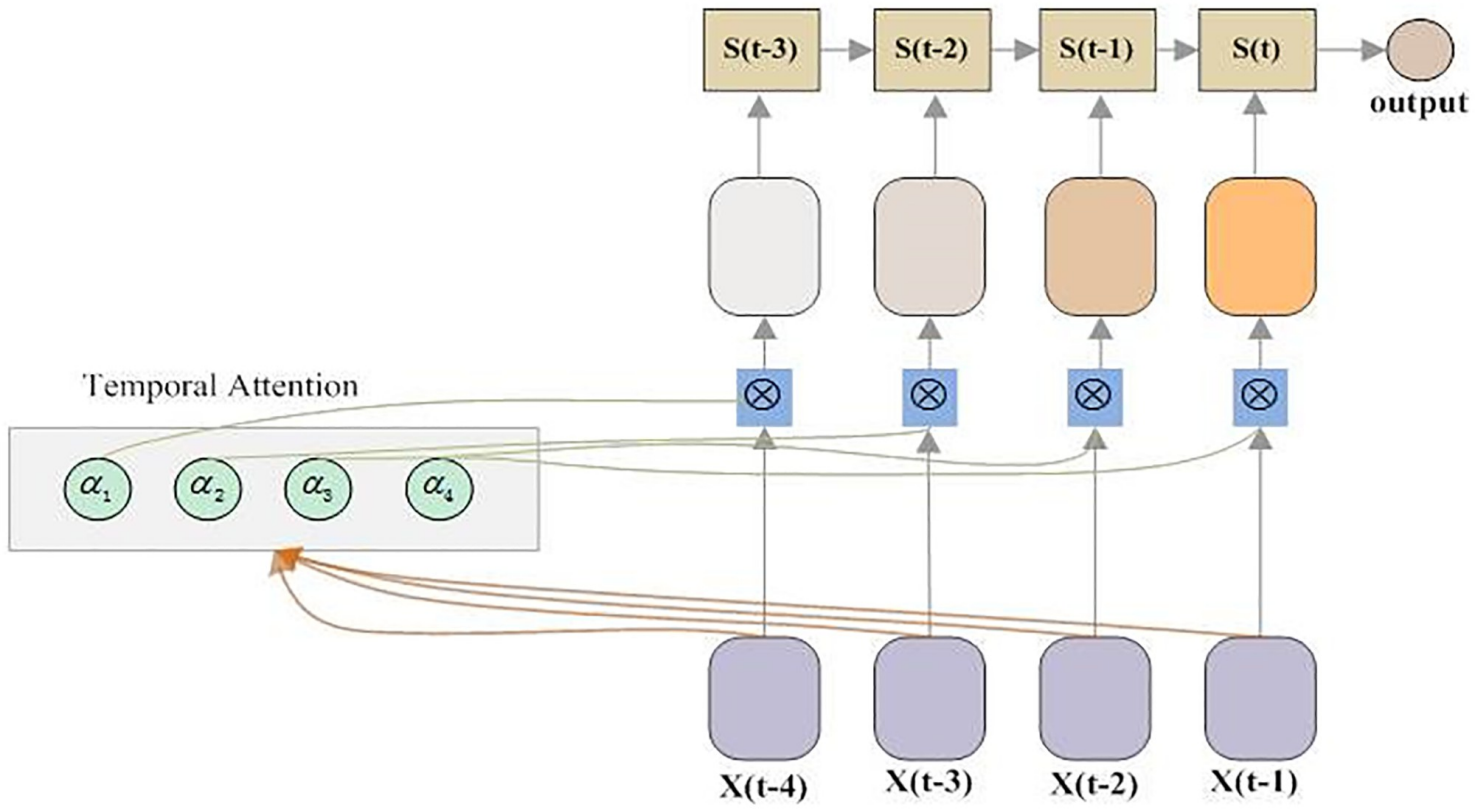
Principle of TA-LSTM working.

In this paper, we propose temporal attention-based LSTM(called TA-LSTM) for multivariate prediction of hepatitis E, based on a hypothesis, the closer the data is to the predicted data, the greater its impact on the results. We employ a linear layer to implement attention computation. The principle is as the following formula (11)–(13). Among them, *e*_*tj*_ means the output of the attention linear layer *a*, and *j* denotes a time step of *T* which is set to 4 in this paper. *x*_*j*_ and *h*_(*t*−1)_ are the input of LSTM at time step *j*. *a*_*tj*_ represents the weight of each time step leveraging softmax function. ${\bar{x}}_{j}$ denotes the weighted input of LSTM. Fig 3 shows the principle of TA-LSTM working. TA-LSTM has the same network structure and parameter settings with the above mentioned LSTM model.

$$\begin{array}{c} e_{tj}=a\left(x_{j},h_{j-1}\right),j\in \left[1,T\right] \end{array}$$ (11)

$$\begin{array}{c} a_{tj}=\frac{exp(e_{tj})}{\sum_{k=1}^{T}exp\left(e_{tk}\right)} \end{array}$$ (12)

$$\begin{array}{c} {\bar{x}}_{j}=\sum_{j=1}^{T}a_{tj}x_{j} \end{array}$$ (13)

#### Multivariate attention

We also propose multivariate attention-based LSTM(called MA-LSTM) for multivariate prediction of hepatitis E, to increase the expression ability of factors with strong correlation and reduce the interference of factors with weak correlation. A linear layer is used to implement the attention distribution. Firstly, we obtain attention score by a learnable linear layer. Then, computing the attention weight by softmax function is needed, as shown in formula (14). *N* denotes the dimension of features. Finally, the new input is obtained by dot product. MA-LSTM also has the same network structure and parameter settings with the above mentioned LSTM model.

$$\begin{array}{c} a_{it}=\frac{exp(e_{it})}{\sum_{k=1}^{N}exp\left(e_{kt}\right)} \end{array}$$ (14)

#### Temporal and multivariate attention

Can we adopt the two attentions (temporal, multivariate attention) to improve the performance of the LSTM? We propose bi-attention-based LSTM (called BiA-LSTM), to verify our ideas. In order to achieve the goal, we first fix the time step attention to train multivariable attention. Then, we alternate the two attention to train again.

### Model evaluation

To verify the effectiveness of the methods, we apply three widely used quality indexes, including Root Mean Square Error (RMSE), Mean Absolute Percent Error (MAPE), Mean Absolute Error (MAE). RMSE is sensitive to the maximum or minimum errors in a group of data, and can well express the precision of measurement, as shown in the formula (15). MAPE is a relative error representation method and is also the most popular indicator for evaluating prediction performance, as shown in the formula (16). MAE shows the actual prediction error, as shown in the formula (17). In the above formulas, *y*_*i*_ and ${\bar{y}}_{i}$ denote true value and predictive value, respectively. *N* denotes the number of samples.

$$\begin{array}{c} RMSE=\sqrt{\frac{1}{n}\sum_{i=1}^{N}{\left(y_{i}-{\bar{y}}_{i}\right)}^{2}} \end{array}$$ (15)

$$\begin{array}{c} MAPE=\sum_{i=1}^{N}\left|\frac{y_{i}-{\bar{y}}_{i}}{y_{i}}\right|\times \frac{100}{N} \end{array}$$ (16)

$$\begin{array}{c} MAE=\frac{1}{N}\sum_{i=1}^{N}\left|y_{i}-{\bar{y}}_{i}\right| \end{array}$$ (17)

## Results

### Results of GRA

In this study, we analyzed the correlation between the monthly incidence, cases of hepatitis E and meteorological factors by GRA, respectively. There are 10 kinds of meteorological data to be analyzed, including maximum temperature(°C), minimum temperature(°C), total rainfall (mm), average temperature(°C), average water vapor pressure(hPa), average minimum temperature(°C), average maximum temperature(°C), days with daily rainfall greater than 0.1mm(days), duration of sunshine(hours), maximum daily rainfall(mm), which are arranged in the order.

**Fig 4:**
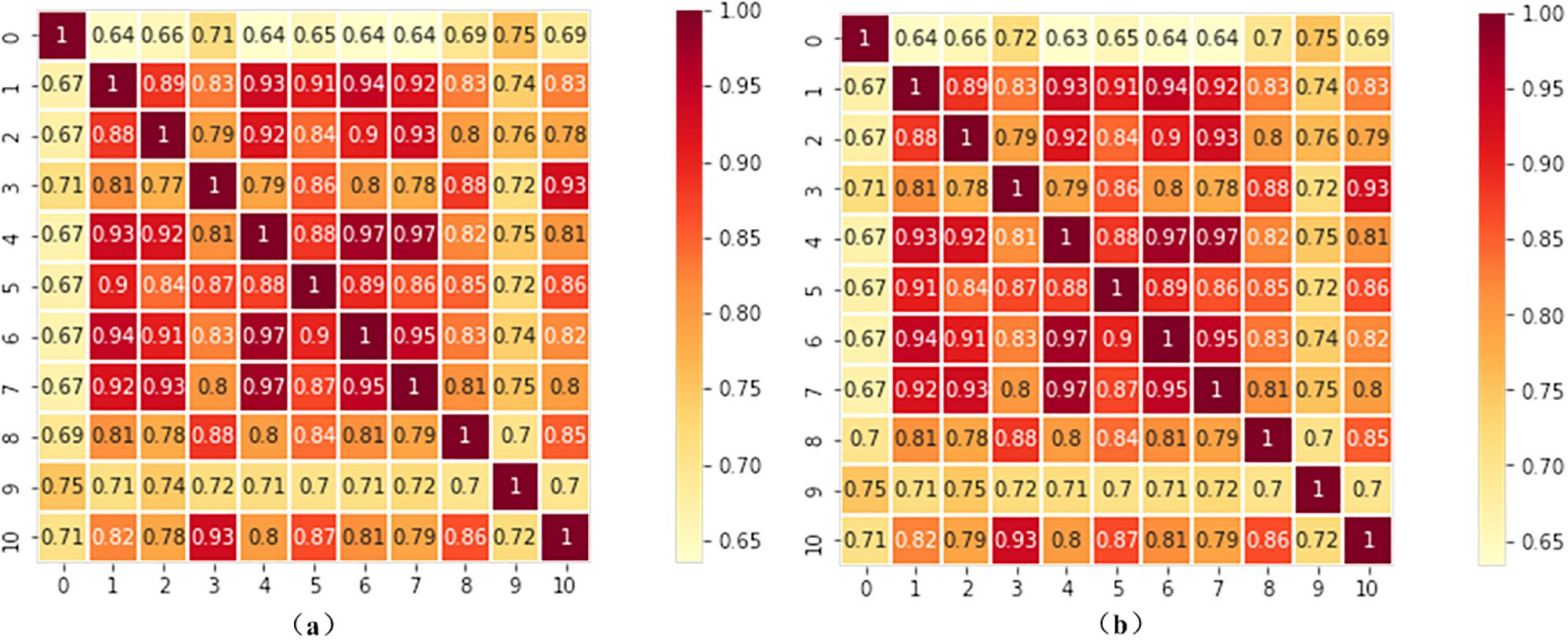
Correlation analysis between the incidence of hepatitis E and meteorological factors. (a) GRA heat map on the incidence of hepatitis E; (b) GRA heat map on the cases of hepatitis E.

The analysis results are displayed in the form of heat map matrix, as shown in Fig 4. Among them, the Fig 4(a) shows the GRA results between the incidence of hepatitis E and meteorological factors, and the Fig 4(b) illustrates the GRA results between the cases of hepatitis E and meteorological factors. The conclusions of the above two analyses are consistent, so we will only discuss the results of Fig 4(b). The meteorological factors relevant to the cases of hepatitis E are duration of sunshine, total rainfall, maximum daily rainfall, days with daily rainfall greater than 0.1mm, respectively. Besides, we can also find that meteorological factors of the same category have strong correlation, such as temperature factors, rainfall factors, etc.

### Results of incidence prediction of hepatitis E

**Fig 5:**
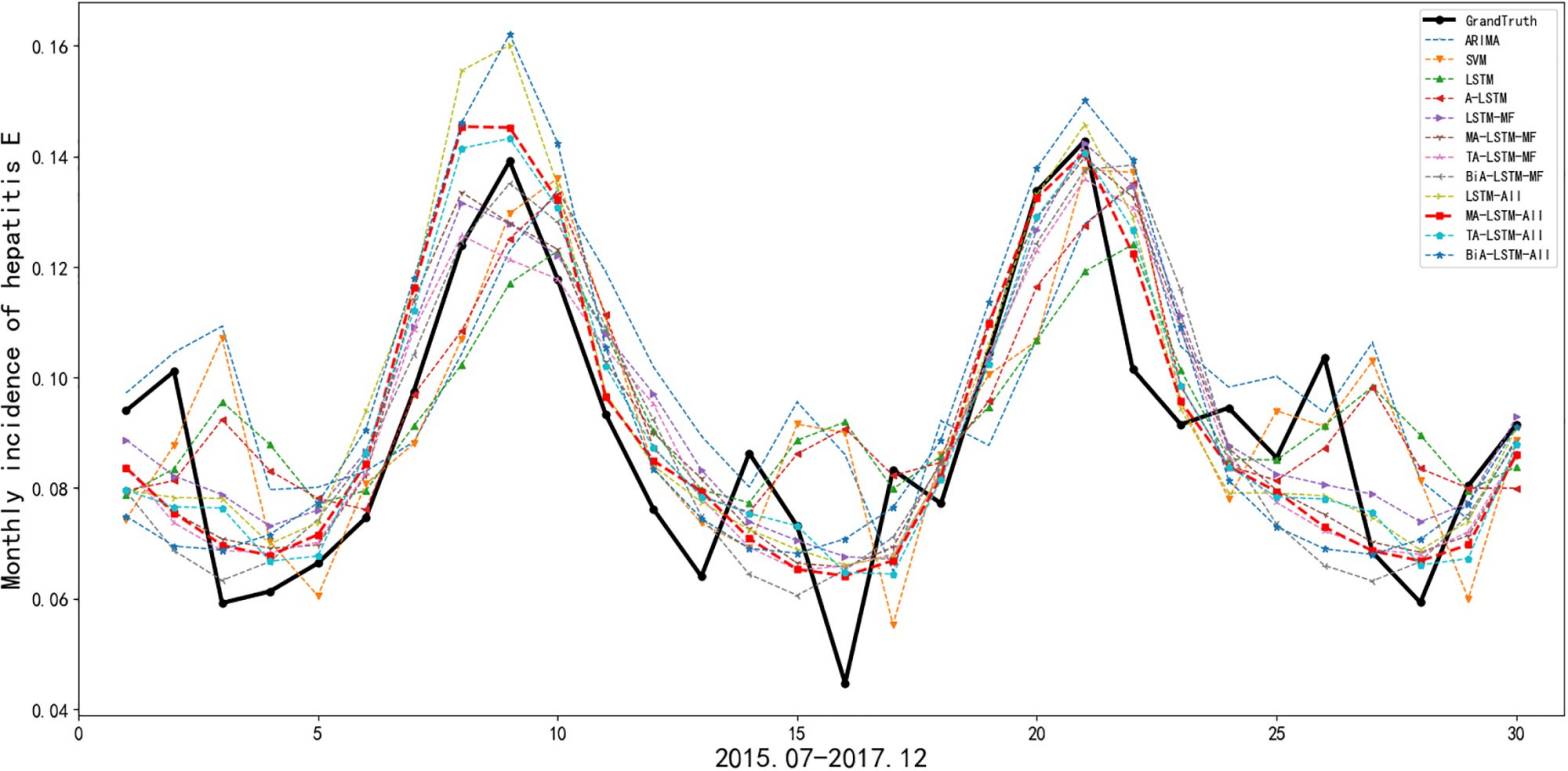
Prediction values of monthly incidence of hepatitis E.

To verify the effectiveness of the methods, we also introduced the experimental results of ARIMA and SVM models, which are derived from our previous work [25]. We divide into two groups of our experiments, namely, univariate prediction experiments(ARIMA, SVM, LSTM and A-LSTM) and multivariate prediction experiments(LSTM-MF, MA-LSTM-MF, TA-LSTM-MF, BiA-LSTM-MF, LSTM-All, MA-LSTM-All, TA-LSTM-All and BiA-LSTM-All), to compare the influence of meteorological factors on the incidence prediction of hepatitis E. The performances of all employed models, in terms of RMSE, MAE, and MAPE, are presented in Table 1. The specific prediction values of hepatitis E incidence in all employed models are illustrated in Fig 5. In these methods, the prefix MA(multivariate attention) represents the multi-variable attention mechanism, and the prefix TA(temporal attention) represents the multi-time attention mechanism. The prefix BiA indicates two attention mechanisms (TA and MA). The suffix MF(main factors) indicates that the method uses several meteorological factors with stronger correlation(total rainfall, days with daily rainfall greater than 0.1mm, duration of sunshine, maximum daily rainfall). The suffix All indicates that all meteorological factors are used in the method.

**Table 1:** Prediction results of hepatitis E incidence by all the models.

Type	Methods	RMSE	MAE	MAPE(%)
Univariate prediction	ARIMA	0.0220	0.0180	23.50
	SVM	0.0204	0.0167	21.70
	LSTM	0.0192	0.0159	20.74
	A-LSTM	0.0181	0.0149	19.50
Multivariate prediction	LSTM-MF	0.0140	0.0117	14.87
	MA-LSTM-MF	0.0135	0.0114	13.84
	TA-LSTM-MF	0.0140	0.0114	13.74
	BiA-LSTM-MF	0.0156	0.0123	14.65
	LSTM-All	0.014	0.012	14.74
	MA-LSTM-All	0.013	0.010	12.91
	TA-LSTM-All	0.013	0.011	13.21
	BiA-LSTM-All	0.0173	0.0145	16.83

For univariate prediction experiments, we can see from Table 1 that A-LSTM achieves the best performance in term of MAPE(19.50%), comparing with ARIMA, SVM, LSTM(23.50%, 21.70%, 20.74%, respectively). Among the methods with main factors, TA-LSTM-MF obtains the best results in term of MAPE by 13.74%, superior to LSTM-MF, MA-LSTM-MF, BiA-LSTM-MF(14.87%, 13.84%, 14.65%). However, among the methods with all meteorological factors, the best model is TA-LSTM-All, which achieves 12.91% in term of MAPE. BiA-LSTM-All method performs the worst(16.83% in term of MAPE) among all the multivariate prediction methods. Comparing LSTM, LSTM-MF, and LSTM-All(20.74%, 14.87%, 14.74% in term of MAPE, respectively), we can find that the more meteorological factors used, the better the prediction effect. When comparing A-LSTM(19.50% in term of MAPE) with MA-LSTM(13.84%, 12.91%, for MF and All, respectively) and TA-LSTM(13.74%, 13.21%, for MF and All, respectively), we can get the same conclusion. However, the conclusion of method BiA-LSTM-MF and method BiA-LSTM-All is an exception, which the all foactor method is inferior to the method with main factors. We will explain the reasons in the discussion section.

Fig 5 presents the predictive monthly incidence of hepatitis E from July 2015 to December 2017 by all the employed methods. It can be seen from Fig 5 that univariate prediction methods(ARIMA, SVM, LSTM and A-LSTM) perform poorly in predicting inflection point, as shown in the middle of Fig 5. The remaining methods are consistent with the trend of grandtruth, especially MA-LSTM-All and TA-LSTM-All methods.

### Results of cases prediction of hepatitis E

**Fig 6:**
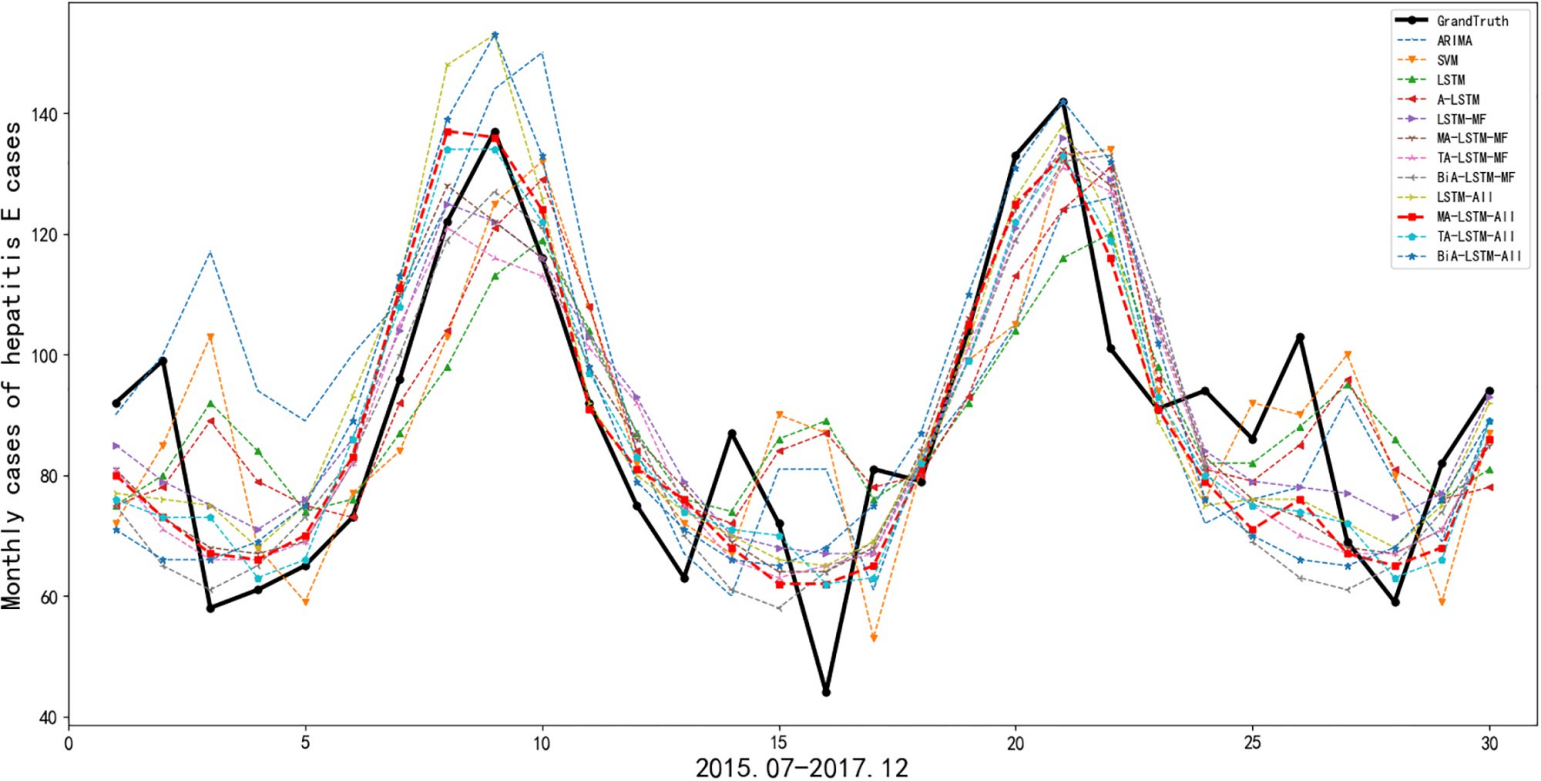
Prediction values of monthly cases of hepatitis E.

Table 2 depicts the evaluation of prediction performance by all the employed methods, in term of RMSE, MAE, and MAPE. The conclusion of this experiment is consistent with that of the previous experiment. A-LSTM is the best model in univariate prediction of hepatitis E cases. MA-LSTM-All is the best in multivariate prediction and all experiments. BiA-LSTM-All also performs poorly in all the multivariate prediction methods. The only difference from the incidence prediction of hepatitis E is that MA-LSTM-MF is better than TA-LSTM-MF. Predictive monthly cases of hepatitis E is shown in Fig 6. It can be seen that the trend of the predictive line generated by MA-LSTM-All is closest to that of grandtruth. Also, the predictive line of MA-LSTM-All is smoother than lines generated by other methods.

**Table 2:** Prediction results of hepatitis E cases by all the models.

Type	Methods	RMSE	MAE	MAPE(%)
Univariate prediction	ARIMA	22.25	18.00	23.60
	SVM	20.04	16.58	21.44
	LSTM	18.75	15.85	20.41
	A-LSTM	17.79	15.06	19.39
Multivariate prediction	LSTM-MF	13.59	11.61	14.77
	MA-LSTM-MF	13.23	11.23	13.59
	TA-LSTM-MF	14.14	11.82	14.12
	BiA-LSTM-MF	15.63	12.64	14.90
	LSTM-All	13.99	11.66	14.20
	MA-LSTM-All	12.65	10.34	12.49
	TA-LSTM-All	12.67	10.59	12.72
	BiA-LSTM-All	16.05	13.18	15.73

## Discussion

Analyzing the related factors of infectious disease is crucial to prevent the spread of infectious diseases. Weather condition is one of the main factors that influence the incidence of diseases. In this paper, we analyzed the correlation between incidence of hepatitis E and 10 meteorological factors, as shown in Fig 4. Duration of sunshine is the most important factor influencing the incidence of hepatitis E, followed by rainfall-related factors, and finally temperature-related factors.

Another idea is whether we can leverage machine learning models to predict the incidence of hepatitis E, to prevent the spread of hepatitis E early. To achieve the above goal, we conducted univariate prediction experiments on hepatitis E incidence data and cases data. The experimental results show that deep learning methods(LSTM, A-LSTM) are superior to the statistical method(ARIMA) and the traditional machine learning method(SVM), due to its powerful feature learning capability. Attention mechanism can improve the performance of LSTM.

We want to implore whether meteorological factors can benefit the prediction performance of hepatitis E. So, we did two groups experiments, one group used the meteorological factors with strong correlation, and the other group used all meteorological factors. Compared with univariate methods, the methods with meteorological factors can greatly improve the prediction performance of hepatitis E incidence(from 19.39% to 12.91%, in term of MAPE). Two sets of multivariate prediction experiments show that meteorological factors with weak correlation are also helpful to improve the prediction capability of the model(from 13.84% to 12.91%, in term of MAPE).

Besides, we propose two types of attention mechanisms, including multivariate attention, temporal attention. The multivariate attention mechanism can distinguish the contribution of each factor to the prediction of the model and carry out weighted processing. As for the temporal attention mechanism, we initially assumed that the closer the location to the predicted value, the greater its impact on the predicted value. Compared with LSTM(14.74%), the two attention-based methods improves to 12.91%, 13.21%, in term of MAPE, respectively. When all meteorological factors are used, MA-LSTM-All(12.91%) is better than TA-LSTM-All(13.21%). This means that the multivariate attention mechanism is more suitable for the prediction of hepatitis E than temporal attention mechanism.

Finally, we try to adopt the two attention mechanisms(BiA-LSTM-All) to improve performance of hepatitis E incidence. Unfortunately, the BiA-LSTM methods perform poorly(14.65%, 16.83%). Meanwhile, BiA-LSTM-All is worse than BiA-LSTM-MF, which is different from other methods. We speculate that this is because our sample is too small, resulting in the inability of model to learn two kinds of attention. In addition, it should be noted that the overall performance of hepatitis E incidence prediction is relatively poor, compared with the prediction in other fields. The reason is that the incidence of hepatitis E is affected by many factors, and the historical data is less. In the future, we will further explore methods to improve the prediction performance of hepatitis E incidence.

## Conclusion

In this study, we analyzed the correlation between the incidence of hepatitis E and meteorological factors. We implemented more than 10 prediction methods using LSTM and attention-based LSTM. The study presents several important conclusions. 1) By the GRA correlation analysis, we found that duration of sunshine and total rainfall are the important meteorological factors affecting the incidence of hepatitis E. 2) Comparing all the employed methods, it can be found that the meteorological data played a positive role in the incidence prediction of hepatitis E and could greatly improve the prediction performance. 3) We can also draw the conclusion that attention mechanism can improve the prediction performance of hepatitis E incidence. 4) Multivariate attention mechanism(MA-LSTM-All) is superior to temporal attention mechanism(TA-LSTM-All) when all meteorological factors are used.

However, we only explored the correlation between the incidence of hepatitis E and meteorological factors, and more detailed research needs to be carried out. In the future work, we will further study the reasons why the dual attention mechanism method(BiA-LSTM) is not effective. In the future work, we will also study how to play the positive role of exogenous data in order to improve the prediction performance of hepatitis E.
